# From case-control averages to validated subgroups: Interpreting inflammatory and neuroaxonal-injury biomarkers in schizophrenia and major depression

**DOI:** 10.1016/j.bbih.2026.101285

**Published:** 2026-06-08

**Authors:** Paul C. Guest, Janet L. Cunningham, Florian Raabe, Kolja Schiltz, Leila Shokati Asl, Thomas Nickl-Jockschat, Johann Steiner

**Affiliations:** a Department of Psychiatry and Psychotherapy, Otto-von-Guericke-University Magdeburg, Magdeburg, Germany; b Laboratory of Translational Psychiatry, Otto-von-Guericke-University Magdeburg, Magdeburg, Germany; c Laboratory of Neuroproteomics, Department of Biochemistry and Tissue Biology, Institute of Biology, University of Campinas (UNICAMP), Campinas, Brazil; d Department of Medical Sciences, Clinical Psychiatry, Uppsala University, Uppsala, Sweden; e Uppsala University Hospital, Psychiatry, Uppsala , Sweden; f Department of Psychiatry and Psychotherapy, Ludwig-Maximilian-University, Munich, Germany; g Max Planck Institute of Psychiatry, Munich, Germany; h Center for Behavioral Brain Sciences (CBBS), Magdeburg, Germany; i Institute of Cognitive Neurology and Dementia Research & Deutsches Zentrum für Neurodegenerative Erkrankungen (DZNE), Magdeburg, Germany; j Departments of Psychiatry, Neuroscience, and Pharmacology, Iowa Neuroscience Institute, University of Iowa, Iowa City, IA, USA; k German Center for Mental Health (DZPG), Partner Site Halle-Jena-Magdeburg, Magdeburg, Germany

**Keywords:** Psychiatric biomarkers, Schizophrenia, Depression, Inflammation, Neuroaxonal injury, Biomarker stratification, Context of use

## Abstract

Psychiatric biomarker research is characterized by subtle but potentially informative biological signals that often lie close to methodological noise. Compared with neurodegenerative or infectious diseases, candidate blood and cerebrospinal fluid (CSF) markers in major psychiatric disorders often show small-to-moderate group differences, substantial patient-control overlap, and a more demanding path to clinical use. Nevertheless, such signals may help identify biologically-meaningful subgroups and contribute to a shift from symptom-based diagnoses toward neuro-immune-endocrine stratification frameworks.

This review examines why such findings occur and summarizes technical sources of noise that can generate false-positive biomarker claims, including limited analytical sensitivity, misinterpretation of assay detection limits, multiplex trade-offs, and pre-analytical factors such as collection timing, season, hemolysis, freeze-thaw cycles, and batch effects. We also discuss how modest effects may become more informative when integrated using validated multivariate approaches.

To calibrate interpretation, we compare psychiatric disorders (e.g., altered neutrophils, cytokines, neurofilament light chain, S100B) with benchmarks from infection and neurodegeneration, and illustrate common pitfalls through laboratory and clinical vignettes. We also outline an analysis-to-synthesis pathway in which biomarkers are evaluated as tools for clinically relevant biological stratification.

Finally, we address publication bias and outcome switching and provide a concise quality checklist for researchers. We argue that progress is more likely to come from prospectively-validated subgrouping strategies than from averaged case-control differences alone, helping move psychoneuroimmunology toward reproducible biomarkers with validated contexts of use.

## 1 Introduction: from weak signals to biological stratification

### 1.1 Weak signals and the psychiatric biomarker problem

This narrative methodological review examines how weak biological signals can be interpreted, validated, and translated into clinically meaningful stratification frameworks. Beyond established cerebrospinal fluid (CSF) biomarkers in the dementias and routine safety monitoring during psychopharmacotherapy, most major psychiatric disorders still lack clinically validated biomarkers for diagnosis, prognosis, or treatment selection in routine practice (Abi-Dargham et al., 2023; Ioannidis, 2005). However, the central challenge may not be the absence of biologically-relevant signals but the mismatch between heterogeneous disorders and diagnostics built primarily around clinical syndromes. Rather than serving as diagnostic tests for broad symptom-defined categories, emerging immune, endocrine, and neurological markers may prove most useful for identifying clinically-relevant subgroups laying the groundwork for reclassification of patient subsets into biologically-defined disease entities.

Unlike many other areas of medicine, psychiatric biomarker research has typically identified modest immune or neurobiological perturbations that are difficult to distinguish from background variability, such that statistically significant case-control differences often show small-to-medium effect sizes, marked distributional overlap, and limited reproducibility (Abi-Dargham et al., 2023). In routine practice, diagnoses such as schizophrenia are established positively based on characteristic clinical syndromes and minimum-duration criteria, while targeted medical work-up (e.g., blood tests, brain imaging, CSF analysis) serves to exclude alternative causes or identify biologically defined subgroups requiring different management. When such work-up is unremarkable, this reflects limitations of currently available methods, not the absence of a biological basis. A longstanding psychiatric tradition from Griesinger (1845) through Kraepelin (1899) to Andreasen (1984) has conceptualized these conditions as brain disorders, and the relative paucity of validated biomarkers reflects methodological difficulty rather than biological absence.

In psychiatric case-control studies, statistically significant mean differences often coexist with wide individual overlap. We therefore distinguish group-effect metrics, including Cohen's d or Hedges' g, from individual-level discrimination metrics, such as the area under the receiver-operating-characteristic curve (AUC) or the proportion of values exceeding a clinically meaningful reference threshold. For example, a medium standardized effect (d = 0.5) corresponds to only modest discrimination (AUC approximately 0.64) and does not support individual classification. Many studies nevertheless discuss group-level effects in language that can be mistaken for individual applicability. This distinction motivates the subgrouping framework developed below.

This dynamic is illustrated by anti-NMDAR encephalitis: Patients may initially present with psychosis but are reclassified once a causal autoantibody is identified (Kayser and Dalmau, 2016; Meng et al., 2016), highlighting how diagnostic boundaries often track mechanistic knowledge.

Diagnostic heterogeneity can further obscure signals. Psychiatric diagnoses are behaviorally defined and biologically diverse (Garcia-Gutierrez et al., 2020). Patients sharing a diagnosis may have distinct pathophysiologies, while different diagnoses may share common features. Whether such overlap reflects shared mechanisms depends partly on the level of analysis. Peripheral inflammatory markers often look similar across disorders on the group level, whereas finer molecular analyses may reveal more specific biological signatures with high relevance for the individual patient. Low-grade inflammation has been reported across major depressive disorder (MDD), schizophrenia, bipolar disorder, and related conditions (Goldsmith et al., 2016; Osimo et al., 2020; Yuan et al., 2019). An umbrella review of 162 peripheral biomarkers across mental disorders found most associations to be underpowered and nonspecific, in the sense that they do not map onto individual diagnostic categories (Carvalho et al., 2020).

Studies often ask whether biomarkers are specific for diagnoses like schizophrenia, although inflammatory markers are altered across many medical conditions and may be more useful for identifying clinically relevant subgroups than diagnostic categories. Progress therefore requires addressing the signal-to-noise problem and the historical ‘organic/non-organic’ split, which may discourage investigation of biologically informative findings in psychiatric patients.

### 1.2 What biomarkers are and what they are not

The U.S. Food and Drug Administration (FDA)–National Institutes of Health BEST Resource defines biomarkers as objectively measured characteristics indicating normal or pathological processes or responses to exposure or intervention, distinct from clinical outcome assessments (Califf, 2018; Group, 2016). In psychiatry, the term is often used without clinical validation. Strictly speaking, biomarkers serve distinct clinical purposes, which matters for interpreting research claims (Califf, 2018; Sechidis et al., 2018):

• Diagnostic (e.g., troponin for myocardial infarction) – no biomarker is currently validated for routine diagnosis of broad, symptom-defined psychiatric syndromes. Instead, new diagnostic groups have been created (e.g., anti-NMDAR encephalitis).
• Prognostic, independent of treatment – some inflammatory markers may have value in psychosis.
• Predictive of treatment response (e.g., HER2 in oncology) – this is largely aspirational in psychiatry.
• Monitoring disease course (e.g., HbA1c in diabetes) – inflammatory markers proposed require validation.
• Pharmacodynamic indicators of biological treatment response (e.g., receptor occupancy).
• Safety biomarkers of toxicity risk - routine psychopharmacologic monitoring includes differential blood count (hematologic effects), liver enzymes and bilirubin (hepatotoxicity), creatinine and estimated glomerular filtration rate (nephrotoxicity), electrocardiography, weight/body mass index (BMI), waist circumference, and fasting glucose and lipids (American Diabetes et al., 2004).

Most psychiatric biomarker studies do not specify intended use. Analytes elevated in patients may represent transient states, stable traits, or unrelated confounding associations. Conflating these contributes to premature claims (Garcia-Gutierrez et al., 2020). Table 1 provides a concise, benchmark-oriented summary of the core markers discussed in the main text, and the **Supplementary Table** provides the expanded evidence and clinical-readiness matrix, including additional widely studied candidates such as brain-derived neurotrophic factor (BDNF).

**Table 1:** Overview of candidate markers discussed, including typical signal magnitude, potential confounders, and current clinical readiness. Note: Effect sizes are reported for meta-analyses, and values are approximate and context-dependent. None of the markers listed are validated for routine diagnostic, prognostic, or predictive use in the psychiatric disorders discussed. Throughout this table, psychiatric signals refer to disorder-level data, whereas benchmark comparators represent more biologically defined conditions.

Marker (matrix)	Domain	Typical psychiatric signal	Benchmark context (large-signal comparator)	Key confounders/pitfalls	Clinical readiness
**Neutrophil count** (blood)	Inflammation (innate)	*Acute psychosis:* FEP 4.70 (3.64–6.26; n = 128) and Sz 4.74 (3.57–6.75; n = 122) vs controls 3.19 (2.62–3.99; n = 293) ×10^9^/L; 23% and 30% above reference vs 6% of controls (Steiner et al., 2020a). Meta-analysis: g = 0.69 overall; larger in first-episode/antipsychotic-naïve cohorts (Dudeck et al., 2025). *Major depression:* FEMD 4.22 (3.43–5.37; n = 81) and RMD 4.52 (3.42–5.39; n = 46) vs controls 3.37 (2.66–4.40; n = 128) ×10^9^/L (Singh et al., 2022). Signal = modest absolute shift with overlap.	*Sepsis/pneumonia:* much larger cell counts. Gram-negative sepsis 13.75 (10.10–20.39; n = 33) and gram-positive sepsis 19.38 (11.41–24.08; n = 18) ×10^9^/L (Sumardi et al., 2021); postoperative pneumonia 9.4 (6.8–13.5) and CAP 9.1 (6.4–12.0) ×10^9^/L (Murakami et al., 2022). Neutrophilia threshold >7.5 × 10^9^/L.	Intercurrent infection, smoking/cigarettes per day, cortisol/stress, obesity/metabolic state, sleep loss, corticosteroids/other medications, and CBC handling all affect counts.	Research-use only; may support inflammatory stratification, not routine psychiatric diagnosis, prognosis, or prediction.
**C-reactive protein** (CRP; blood)	Inflammation (acute-phase)	Meta-analytic elevation but still low-grade in absolute terms. *Schizophrenia:* g = 0.60 overall (drug-naïve/free g = 0.87; first episode g = 0.63; chronic g = 0.76) (Fernandes et al., 2016). Representative medians in acute psychosis remain low-grade: FEP 2.1 (0.6–4.0; n = 128), Sz 3.1 (1.0–4.0; n = 121) vs controls 1.0 (0.5–2.1; n = 294) mg/L (Steiner et al., 2020a). *Major depression:* g = 0.71 in a larger recent meta-analysis (Osimo et al., 2020) and d = 0.47 overall/d = 0.69 in the high-quality subset of the cumulative meta-analysis (Haapakoski et al., 2015); FEMD 3.61 (1.0–4.0; n = 82), RMD 2.00 (1.0–4.0; n = 45) vs controls 1 (1–3; n = 129) mg/L (Singh et al., 2022). High overlap; values typically remain in the low-grade range.	Acute *bacterial inflammation/sepsis* usually exceeds psychiatric values by ≥ 1 order of magnitude; in severe sepsis, CRP extends into the hundreds of mg/L (up to ∼400–500 mg/L) (Singh et al., 2025).	BMI/waist circumference, smoking, infections, medical comorbidity/metabolic syndrome, season/sample timing, and analytic method.	Research-use only; potential inflammatory stratifier, but not validated for routine psychiatric diagnosis, prognosis, or prediction.
**Interleukin-6** (IL-6; blood/CSF)	Inflammation (cytokine)	Most consistent cytokine signal. Acute meta-analytic ESs: schizophrenia 1.16 (first episode) and 0.73 (acute relapse), bipolar mania 0.59; MDD estimates vary by synthesis/inclusion criteria: 0.76 in acute MDD (Goldsmith et al., 2016), g = 0.61 overall (Osimo et al., 2020), and d = 0.54 overall/d = 0.60 high-quality subset (Haapakoski et al., 2015). After treatment IL-6 falls modestly (ES change ≈−0.13 to −0.36). Chronic states are smaller: schizophrenia 0.27, euthymic bipolar 0.30, MDD 0.39 (Goldsmith et al., 2016; Haapakoski et al., 2015). CSF findings are less consistent and limited by low concentrations and assay sensitivity (Singh et al., 2023).	Sepsis is orders of magnitude larger: median IL-6: 788 pg/mL without shock and 10,049 pg/mL with septic shock; peaks up to 500,000 pg/mL (Damas et al., 1992).	Also affected by BMI, infections, smoking, diurnal/seasonal effects, and processing delays. Very strong assay/LLOQ dependence in CSF.	Research-use only; mechanistic/subgrouping marker, without validated psychiatric cutoffs.
**Tumor necrosis factor-α** (TNF-α; blood)	Inflammation (cytokine)	More modest and less stable than IL-6. Acute meta-analytic ESs: schizophrenia 0.31 (first episode) and 0.22 (acute relapse), bipolar mania 0.43; acute MDD estimates vary across meta-analyses and inclusion criteria (∼0.35–0.54 overall; weaker/non-significant in high-quality subsets): 0.35 in acute MDD (Goldsmith et al., 2016), g = 0.54 overall (Osimo et al., 2020), and d = 0.40 overall/d = 0.28 high-quality subset [p = 0.09, NS ](Haapakoski et al., 2015). Chronic schizophrenia ES 0.30; chronic MDD shows no significant mean difference (ES 0.05) (Goldsmith et al., 2016; Haapakoski et al., 2015). Associations with symptoms/treatment response remain inconsistent. CSF findings are less consistent and limited by low concentrations and assay sensitivity (Singh et al., 2023).	Autoimmune and infectious conditions produce substantially larger circulating TNF-α concentrations than psychiatric cohorts, reinforcing that psychiatric effects occur at the low end of the assay range.	BMI/obesity, smoking, medications, and comorbid inflammatory/autoimmune disease can dominate observed differences. Low-abundance analyte; platform/multiplex variability. Very strong assay/LLOQ dependence in CSF.	Research-use only; hypothesis-generating and subgroup work, not routine psychiatric diagnosis, prognosis, or prediction.
**Neurofilament light chain** (NfL; blood/serum) *Note: CSF NfL evidence is more robust and should be interpreted separately*	Neuroaxonal injury	Blood NfL in primary psychiatric cohorts is usually single-digit to low-teens pg/mL and often overlaps controls (Bavato et al., 2024). TRS schizophrenia: 6.3 [5.5, 7.2] pg/mL (n = 82) vs controls 5.8 [5.3, 6.3] (n = 58); non-clozapine schizophrenia 4.9 [4.0, 5.8] (n = 13) (Eratne et al., 2022). In an older differential-diagnosis cohort: schizophrenia 11.6 (9.8–23.5; n = 11), depression 15.7 (12.4–25; n = 28), bipolar 17.8 (12.6–23.1; n = 11), controls 15.1 (11.3–19.1; n = 27) (Al Shweiki et al., 2019). CSF NfL has the more established evidence base overall (Bavato et al., 2024).	bvFTD: 72.7 (28.3–90; n = 20) pg/mL – roughly 4–6× the psychiatric cohorts (Al Shweiki et al., 2019).	Strong age dependence; additional influence of BMI, renal function, neurological comorbidity/head injury, and assay platform/matrix differences.	Potential differential-diagnosis adjunct when neurodegeneration is suspected; not validated for routine psychiatric diagnosis, prognosis, or prediction.
**S100B** (blood)	Glial integrity/barrier/metabolic confounding	Earlier summaries suggested elevated peripheral S100B across schizophrenia and mood disorders, but heterogeneity is large and disease specificity is poor (Schroeter and Steiner, 2009); a more recent systematic review across 111 studies in five major psychiatric disorders found that most studies reported elevation, but some reported no difference or decreases (Kozlowski et al., 2023). Representative serum concentrations are usually only modestly higher than controls (∼0.05 μg/L in controls vs ∼0.07 μg/L in schizophrenia cohorts), and interpretation is heavily confounded by BMI/insulin resistance and other peripheral sources (Steiner et al., 2010a, 2010b).	MCA stroke day 2 serum: favorable outcome (mRS 0–1) median 0.10 (IQR 0.07–0.14; n = 60) μg/L, unfavorable outcome (mRS 2–6) 0.20 (IQR 0.11–0.48; n = 109) μg/L, i.e. ∼2–5× normal (Luger et al., 2021). Severe TBI: mean ∼1.2–4.9 μg/L, i.e. up to ∼100× normal vs. controls ∼0.04–0.05 μg/L; positive correlation with injury volume and clinical outcome (Rothermundt et al., 2003).	BMI/insulin resistance, peripheral expression (adipose tissue, gastrointestional, lymphocytes), hemolysis, blood–brain barrier status, assay detection limits in the low range, seasonal and day/night variation.	Exploratory; useful cautionary example of confounding rather than a validated clinical marker.
**Weight/BMI, glucose, lipids** (blood)	Safety monitoring (treatment-related)	Antipsychotics produce clinically meaningful metabolic changes. Estimated weight gain at 10 weeks vs placebo: +0.5 kg (ziprasidone) to +4.5–5.0 kg (clozapine/olanzapine). Clozapine and olanzapine carry highest risk for diabetes and dyslipidemia; aripiprazole and ziprasidone the lowest (American Diabetes et al., 2004). Clinical guidelines recommend routine monitoring (fasting glucose, lipid profile, waist circumference; ADA/APA consensus monitoring protocol) (American Diabetes et al., 2004).	Not applicable (treatment safety markers, not disease markers).	Implementation/adherence issues; baseline metabolic risk; fasting status for lipids/glucose; medication selection; polypharmacy; real-world monitoring uptake often incomplete.	Routine, guideline-recommended safety monitoring during antipsychotic therapy, though implementation remains incomplete in routine care.

**Abbreviations:** FEP, first-episode psychosis; Sz/SCZ, schizophrenia; FE-MDD, first-episode major depression; RMD, recurrent major depression; CAP, community-acquired pneumonia; CBC, complete blood count; CRP, C-reactive protein; g, Hedges' g; d, Cohen's d; MDD, major depressive disorder; BMI, body mass index; IL-6, interleukin-6; CSF, cerebrospinal fluid; ES, effect size; LLOQ, lower limit of quantification; CLSI, Clinical and Laboratory Standards Institute; TNF-alpha, tumor necrosis factor-alpha; NS, not significant; RA, rheumatoid arthritis; CI, confidence interval; NfL, neurofilament light chain; TRS, treatment-resistant schizophrenia; bvFTD, behavioral variant frontotemporal dementia; S100B, S100 calcium-binding protein B; BD I, bipolar disorder type I; MCA, middle cerebral artery; mRS, modified Rankin Scale; IQR, interquartile range; TBI, traumatic brain injury; ADA, American Diabetes Association; APA, American Psychiatric Association.

This review focuses on exploratory-phase biomarkers, where translation from case-control difference to clinical utility requires analytical validation, replication, biologically-informed subgrouping, prospective testing, and demonstration of clinical utility (Califf, 2018; Group, 2016). We argue that the most informative role of many psychiatric biomarkers at this stage may be biological stratification rather than diagnosis of broad symptom-defined syndromes. The path from exploratory association to clinical implementation is sequential: analytical validation, replication, biologically informed subgrouping, prospective testing, and demonstration of clinical utility. The point is not to replace symptom-guided treatment but to refine it. This staged pathway is summarized in Fig. 2.

Accordingly, candidate markers should be linked to explicit Context-of-Use (CoU) statements that define the biomarker category, target population, clinical decision, and intended action. In this review, realistic near-term CoU examples include the use of NfL as a differential-diagnostic or rule-out adjunct when psychosis presents with atypical cognitive or neurological features, and the use of CRP/IL-6 as predictive-enrichment markers in trials of immune-modulating interventions. These examples are not proposed as stand-alone diagnostic tests for schizophrenia or major depression.

### 1.3 From weak signals to biological stratification

Discerning signal from noise is a central challenge in psychiatric biomarker research because reported effects are often smaller than those observed in infection, autoimmunity, or neurodegeneration, yet may still contain clinically meaningful information. Distinguishing methodological artefact, nonspecific physiology, and genuine stratification signals therefore requires rigorous study design, attention to analytical limitations, and willingness to revise interpretations when results contradict expectations (Feynman, 1974). No downstream analysis can compensate for inadequate group matching, absent preregistration, or uncontrolled confounders.

This review examines why biologically-relevant signals in psychiatry often appear weak relative to methodological noise and how they may nevertheless contribute to clinically-meaningful stratification. We compare psychiatric biomarker readouts with those from infection and neurodegeneration, examine sources of pre-analytical and analytical variability, and illustrate common pitfalls through laboratory and clinical vignettes. Throughout, biomarker development is framed as an iterative process that cycles between analysis of individual signals and synthesis into biologically-coherent subgroups (Fig. 2). The review also provides practical tools for distinguishing robust findings from artefact and prioritizing reproducibility over premature biomarker claims.

### 1.4 What this review adds

**Fig. 1:**
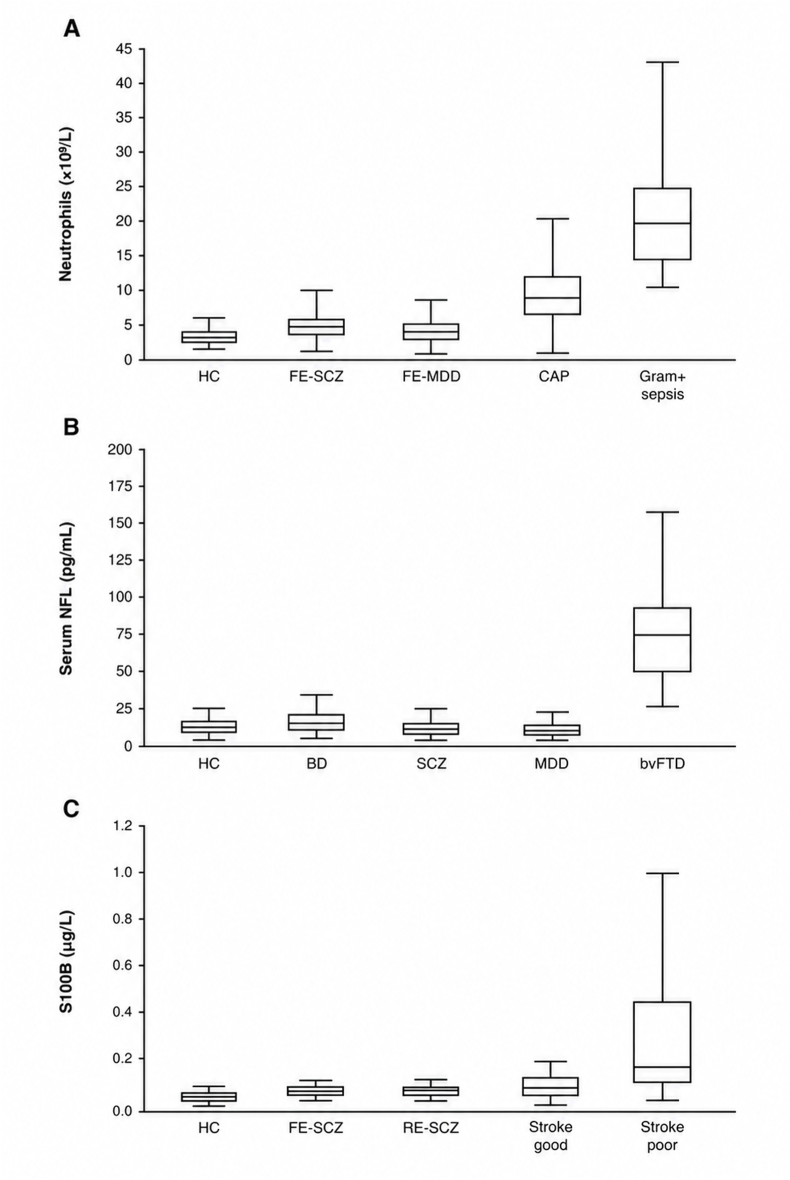
Signal in context: biomarker shifts in psychiatric disorders on the group level tend to be relatively small and show substantial overlap with healthy controls, compared with conditions defined by clearer biological mechanisms (e.g., infection or neurodegeneration). (A) Neutrophil counts in healthy controls (HC; n = 293), first-episode schizophrenia (FE-SCZ; n = 128), and first-episode major depressive disorder (FE-MDD; n = 81) contrasted with community-acquired pneumonia (CAP; n = 1027) and Gram-positive sepsis (n = 18) benchmarks (Murakami et al., 2022; Singh et al., 2022; Steiner et al., 2020a; Sumardi et al., 2021). (B) Serum neurofilament light chain (NfL) in healthy controls (n = 27), bipolar disorder (BD; n = 11), schizophrenia (SCZ; n = 11), major depressive disorder (MDD; n = 28), and behavioral-variant frontotemporal dementia (bvFTD; n = 20) (Al Shweiki et al., 2019). (C) Serum S100B in healthy controls (n = 22), first-episode schizophrenia (n = 11), and recurrent schizophrenia (n = 6) (Milleit et al., 2016), and acute ischemic stroke with favorable (mRS 0-1; n = 60) versus unfavorable outcome (mRS 2-6; n = 109) (Luger et al., 2021). Note: Panels A-C use published data from different studies and assay platforms; they are illustrative benchmark comparisons, not head-to-head measurements.

**Fig. 2:**
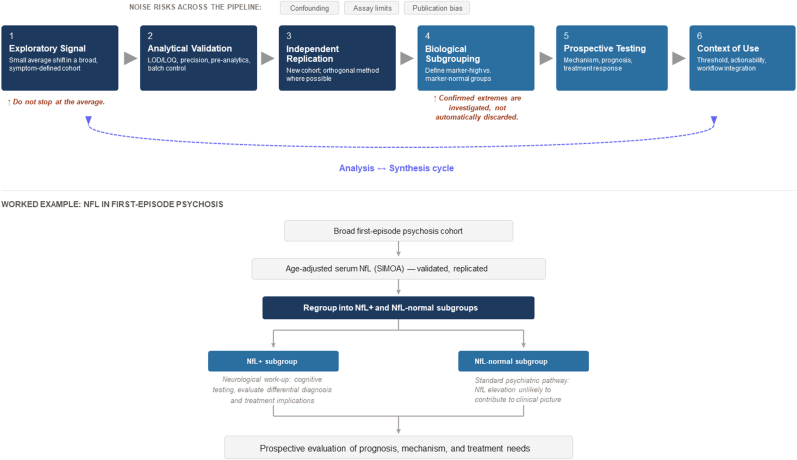
Conceptual pathway from exploratory biomarker signals to clinically useful subgroup markers in psychiatry. Biomarker development should proceed from initial exploratory observations in broad, symptom-defined cohorts through analytical validation and independent replication, followed by biologically informed subgrouping rather than reliance on average case–control differences alone. Confirmed extreme values are investigated as potential subgroup-defining signals rather than discarded automatically. Prospectively testing whether such subgroups differ in mechanism, prognosis, or treatment response is a prerequisite for establishing context of use and eventual implementation. The dashed feedback arrow indicates that the process is iterative and cycles between analysis of individual signals and synthesis into biologically meaningful clinical models. The illustrative use case is conceptual and does not report primary cohort data. It does show how an age-adjusted NfL result in first-episode or atypical psychosis could support differential-diagnostic triage and prospective subgroup testing, rather than diagnosis of schizophrenia. Note: In FDA-NIH BEST terminology, translation requires clearly defined Context of Use (CoU) statements specifying the biomarker intended role and decision context. Depending on the application, biomarkers may function as prognostic or predictive markers (Califf, 2018; Sechidis et al., 2018). Regulatory qualification through frameworks such as the FDA Biomarker Qualification Program or the European Medicines Agency qualification of novel methodologies requires analytical validation, clinical validation in independent cohorts, and prospective demonstration of clinical utility for the specified CoU. In psychiatry, realistic near-term targets may include NfL as a differential-diagnostic or rule-out adjunct for neurodegenerative or neurological disease, and inflammatory markers such as CRP as predictive enrichment biomarkers for trials of immune-modulating treatments.

Building on prior reviews that catalogued inflammatory signals and biomarker-validation gaps (Abi-Dargham et al., 2023; Carvalho et al., 2020; Goldsmith et al., 2016), this review contributes four elements not emphasized in previous overviews: (i) benchmark calibration against infection, autoimmunity, and neurodegeneration (Fig. 1); (ii) a decision-based pathway linking biomarker findings to subgrouping and prospective validation (Fig. 2); (iii) integration of pre-analytical and assay-related failures with laboratory vignettes; and (iv) consideration of how successful biomarker-based stratification may contribute to biologically-characterized disease reclassification.

This is a narrative methodological review, not a systematic review or meta-analysis. Quantitative estimates were drawn preferentially from published systematic reviews, meta-analyses, regulatory resources, and exemplar trials. Additional illustrative examples were identified through targeted, non-systematic searches of PubMed and reference lists up to June 2026. The synthesis is therefore intended to calibrate interpretation and define translational logic, rather than to provide an exhaustive evidence map. Some laboratory vignettes derive from the authors’ own work and are explicitly identified as such.

## 2 Analytical sensitivity and detection limits

While single-analyte sensitivity and specificity remain foundational (multi-marker approaches in Section 2.3), many assays were developed for conditions with large biological changes. Weak biological effects in single-analyte measures are therefore difficult to separate from noise. Careful evaluation of analytical sensitivity, detection limits, and assay validation is essential before interpreting small effects as meaningful.

### 2.1 Ultra-sensitive assays for low-level biomarkers in blood

Until recently, most brain-derived proteins were virtually undetectable in blood at the low concentrations typically observed in psychiatric patients. NfL, a cytoskeletal protein released during neuroaxonal injury, rises significantly in neurodegenerative diseases (Bavato et al., 2024). In CSF, standard ELISA platforms have allowed identification of neuroinflammatory conditions. In contrast, blood NfL differences in psychiatric disorders are subtle. Only ultra-sensitive immunoassays such as single molecule array (SIMOA) can quantify the minute fluctuations reported in schizophrenia and related conditions (Bavato et al., 2024). Even then, these differences are only a few picograms per millilitre at the fringe of detectability, making rigorous validation essential. Although measurement in serum is convenient, these platforms can be costly.

### 2.2 Limits of detection and quantification

When interpreting weak assay signals, it is critical to distinguish between limit of detection (LOD), the lowest concentration distinguishable from zero, and limit of quantification (LOQ), the lowest concentration that can be measured with acceptable precision (Andreasson et al., 2015). Importantly, the coefficient of variation (CoV) of most immunoassays increases sharply near the LOQ, meaning that the concentration range relevant to psychiatric biomarker studies is also the range where measurement imprecision is greatest.

In many psychiatric cytokine studies, reported concentrations fall at or below the LOQ, rendering them indistinguishable from noise. This applies to both CSF and serum/plasma analytes. Accordingly, in a panel of cytokines measured in schizophrenia patient CSF, most were found to be undetectable or barely above the LOD (Singh et al., 2023). Rather than over-interpreting, the authors reported these limits transparently, exemplifying rigor through negative findings, as detailed by Niculescu et al. (2015). When reporting biomarker data, the percentage of cases with values near or below the LOD and/or LOQ should be disclosed, along with the imputation methods used, as different strategies (e.g., LOD/2, LOD/√2, or multiple imputation) can materially alter group comparisons when a substantial proportion of values fall below these limits (Herbers et al., 2021). If values cluster near or below the LOQ, accepting a null result rather than forcing significance through aggressive imputation or threshold adjustment reflects both scientific integrity and good analytical practice.

### 2.3 Multiplex assays: convenience at a cost

Multiplex assays allow simultaneous measurement of dozens of analytes but often at the expense of sensitivity, accuracy, or dynamic range (de Koning et al., 2012). Many multiplex immunoassays have higher LODs for individual analytes than the corresponding single-analyte assays. Testing multiple analytes also increases the risk of spurious positives without use of multiple-comparison correction, such as controlling for the false discovery rate (FDR) (Menyhart et al., 2021).

Choosing the appropriate method requires balancing study goals with practical constraints. Traditionally, this has meant focusing on a few well-justified analytes using the most sensitive assays available. However, systems immunology and modern machine-learning approaches are increasingly capable of handling larger proteomic and metabolomic datasets as integrated systems, clustering analytes by biological pathways rather than treating them as isolated variables. This can add considerable robustness to interpretation, particularly when groups are defined by biological variables rather than symptom-based psychiatric diagnoses. When used in this manner, multi-omics profiling with integration of transcriptomic, proteomic, and metabolomic layers, may prove more informative than any single analyte, since psychiatric pathophysiology is unlikely to reduce to a single molecular signal.

However, it should be noted that multi-marker approaches do not solve the signal-noise problem automatically as within-sample accuracy can reflect overfitting.

### 2.4 Assay controls, replication and method choice

Working with weak signals requires rigorous controls and replication. The inclusion of blinded duplicate samples within and between batches can reveal whether variability reflects biological differences or assay instability (Valentin et al., 2011). Large discrepancies between duplicates should cast doubt on subtle group differences. Positive control samples (e.g., serum from individuals with acute infection or autoimmune disease) should confirm that assays perform accurately across the dynamic range and consistently between batches (Valentin et al., 2011). In multicenter studies, systematic site effects can introduce variance that mimics or masks group differences and can be addressed through quality-control samples across sites and by modeling site as a covariate. Such quality-control steps are routine in fields like endocrinology but are sometimes neglected in exploratory psychiatric biomarker studies, especially those under publication pressure (Abi-Dargham et al., 2023). Extreme values should not be discarded automatically. They should first trigger assay verification and review of pre-analytical factors. If confirmed, they should be examined as candidate biological subgroups and reported transparently.

Finally, assay choice can determine accuracy of measurement, as illustrated by NMDAR autoantibody measurement in psychiatric patients (**Vignette 1**). Different assay formats can yield strikingly discordant results due to differences in analytical sensitivity at low titers, or because they may target different epitopes or employ different cell fixation methods. A finding can therefore appear robust using one research platform while being undetectable on another, even when both assays are technically performing within their own specifications.

## 3 Pre-analytical pitfalls and hidden confounders

Even when appropriate assays are applied in biomarker studies, upstream sample collection and handling can generate spurious signals. These pre-analytical factors are often overlooked, yet can obscure weak biological effects, especially in multicenter studies.

### 3.1 Sample collection variables

The concentration of many blood biomarkers is influenced by collection conditions. Cytokine levels can vary with feeding status, reflecting metabolic and hormonal changes, and follow diurnal rhythms linked to immune regulation. In females, immune and hormone markers fluctuate across the menstrual cycle (Oertelt-Prigione, 2012; Whitcomb et al., 2014), and both sexes show higher inflammatory markers (e.g., IL-6, CRP) and neutrophil counts in winter versus summer, likely due to increased infections, hormonal variations, and reduced sunlight (Dopico et al., 2015; Liu and Taioli, 2015). Therefore, if psychiatric patients are recruited in winter and controls in summer, an apparent case-control difference in IL-6 or CRP could reflect seasonal variation unrelated to pathology.

### 3.2 Handling and processing artifacts

Post-collection handling can introduce additional noise. Delayed processing, centrifugation differences, or storage temperature variations can distort biomarker signals (Betsou et al., 2010). Hemolysis from difficult venipuncture or poor handling can rupture red blood cells, causing leakage of intracellular contents and altering levels of multiple analytes, including cytokines (Marques-Garcia, 2020). If samples from one group are more frequently hemolyzed (e.g., patients who were harder to draw or whose samples sat longer before processing), spurious group differences may emerge. Freeze-thaw cycles degrade some biomarkers, so unequal handling across groups (e.g., one thaw versus two) can create artificial differences (Lee et al., 2015; Mitchell et al., 2005). As illustrated in **Vignette 2**, even a statistically robust biomarker signal can result from an unrecognized systemic confounder such as BMI rather than brain pathology.

The solution is to standardize pre-analytical protocols in accordance with biobanking best practices (Betsou et al., 2010; Vaught, 2016). Including quality-control samples across the pipeline can help to detect site- or laboratory-specific artifacts. Also, pre-analytical variables should be recorded in the dataset and treated as potential confounders in the analysis.

## 4 Signal in context: calibrating scientific judgment

The comparisons below are not intended to show that psychiatric markers “fail” because they show smaller effects than markers in infection or neurodegeneration, but to clarify why broad diagnostic averaging can obscure clinically meaningful subgroups. As noted in Section 1.1, these comparisons are also partly circular. Conditions defined by biological criteria will inherently show larger and more consistent biomarker effects than symptom-defined syndromes, and findings that contribute to biological reclassification further widen the apparent separation between fields.

Groups selected based on shared molecular patterns or pathophysiology will be more homogeneous than those defined by symptom patterns. The goal of subgroup-based biomarker research is to identify biological signatures within these that may correspond to disease-relevant mechanisms.

We present single-marker examples here because interpretive competence begins with understanding individual analytes. Understanding their reference ranges, confounders, and dynamic behavior is a prerequisite for the multi-marker and systems-level approaches discussed in Section 2.3.

### 4.1 Neutrophil counts: psychosis and major depression versus infection

A meta-analysis of 26,349 schizophrenia patients and 16,379 controls reported significantly elevated neutrophil counts at the group level (Hedges' g = 0.69), with larger group effects in first-episode (g = 0.85) and antipsychotic-naïve (g = 1.17) patients (Dudeck et al., 2025). Similar, albeit more modest, elevations were reported in first-episode and recurrent MDD (Singh et al., 2022).

However, these group-level statistics obscure a more informative pattern. In our studies, 23% of first-episode and 30% of relapsed schizophrenia patients exceeded the reference range, compared to 6% for controls (Steiner et al., 2020a). Median values remained within normal limits (schizophrenia, 4.70 × 10^9^/L; depression, 4.2–4.5 × 10^9^/L) with no clear separation from controls (Singh et al., 2022). Crucially, the majority of patients did not show elevations despite significant groupwise differences, indicating that a subgroup with values above the reference was driving the statistical signal.

In contrast, pneumonia (Murakami et al., 2022) and sepsis (Sumardi et al., 2021), both biologically defined conditions, commonly produce neutrophil counts of ∼9–20 × 10^9^/L, two to four times higher than those observed in psychiatric cohorts (Fig. 1A). Expecting comparable effect sizes from broad, symptom-defined psychiatric disorders is therefore unreasonable.

Rather than dismissing mild neutrophil elevations as epiphenomena, stratifying patients into those with and without values above reference and asking how these subgroups differ clinically may prove more productive. Even a minority of patients with elevated counts may represent the biologically most informative subset.

### 4.2 NfL and S100B: psychiatric disorders in the context of neurologic benchmarks

As a marker of neuroaxonal injury, NfL shows robust elevations in CSF and blood in biologically defined diseases (Alzheimer's disease, frontotemporal dementia, amyotrophic lateral sclerosis), where levels often correlate with disease severity (Khalil et al., 2024). For example, a SIMOA study demonstrated clear separation between patients with behavioral-variant frontotemporal dementia and controls with several-fold higher serum NfL levels in the disease group (Al Shweiki et al., 2019). Similar large elevations are observed in Alzheimer's disease and amyotrophic lateral sclerosis (Lu et al., 2015; Mattsson et al., 2017).

In contrast, in symptom-defined psychiatric disorders such as schizophrenia, bipolar disorder, or MDD, blood NfL levels generally remain in the single-digit to low-teens pg/mL range and substantially overlap with controls, particularly after age adjustment (Bavato et al., 2024). Across published studies, blood NfL findings in primary psychiatric disorders are small, age-sensitive, heterogeneous, and substantially overlapping with control distributions, whereas larger elevations are observed in neurodegenerative diseases (Lu et al., 2015; Mattsson et al., 2017). Interpretation of blood NfL findings in psychiatric disorders is further complicated by strong age dependence and additional influences such as BMI, renal function, neurological comorbidity, or head injury (Bavato et al., 2024) (Fig. 1B).

S100B shows a similar pattern in psychiatric cohorts showing only modest serum shifts (∼1.5–2× above normal), whereas acute ischemic stroke raises serum S100B to ∼2–5× normal and severe traumatic brain injury can raise it by up to ∼100-fold, depending on lesion burden and outcome (Kozlowski et al., 2023; Luger et al., 2021; Rothermundt et al., 2003) (Fig. 1C).

These examples show that biomarkers in psychiatric disorders are often closer to normal reference values and are unlikely to function as stand-alone diagnostic cutoffs, unlike those in biologically defined conditions. Instead, their clinical value is more likely to lie in identifying subgroups with distinct biology, clinical trajectories, or diagnostic needs. For example, markedly elevated serum NfL in a first-episode psychosis patient may serve as a red flag prompting diagnostic work-up to exclude neurodegeneration or other neuroaxonal injury, or to inform prognosis (Al Shweiki et al., 2019; Eratne et al., 2024). Fig. 2 illustrates this approach. Similarly, the strong correlation of S100B with BMI and insulin resistance (Steiner et al., 2010a, 2010b) shows how understanding peripheral confounders can redirect clinical attention toward metabolic comorbidity, a key determinant of morbidity and mortality in severe mental illness.

### 4.3 Evidence that biological stratification can guide treatment

In MDD, evidence for biomarker-based stratification is already emerging. In treatment-resistant depression, infliximab showed no overall benefit, but patients with baseline CRP >5 mg/L improved significantly more than those receiving placebo, whereas patients with lower CRP did not benefit (Raison et al., 2013). Similarly, the GENDEP study found that CRP predicted differential response to antidepressant class, with higher CRP favoring nortriptyline over escitalopram (Uher et al., 2014).

A similar pattern has been observed in schizophrenia. Trials targeting inflammatory pathways, including tocilizumab and other anti–IL-6 approaches, have generally been negative in unselected cohorts but have raised the possibility of benefits in inflammatory subgroups (Girgis et al., 2018; Miller et al., 2016). Also, meta-analyses of anti-inflammatory augmentation have reported modest and heterogeneous effects, consistent with the idea that immune mechanisms are clinically relevant in a subset of patients (Köhler-Forsberg et al., 2019).

These studies do not yet establish clinically validated biomarkers. However, they demonstrate that biological stratification can generate treatment-relevant signals that are obscured when heterogeneous patient populations are analyzed as single diagnostic groups.

### 4.4 Cytokines and acute phase proteins

Meta-analyses show that IL-6 is among the most consistent inflammatory signals in psychiatric disorders, with the highest levels observed in first-episode schizophrenia, followed by acute relapsed schizophrenia, bipolar mania, and acute MDD (Goldsmith et al., 2016; Haapakoski et al., 2015; Osimo et al., 2020). TNF-alpha effects are smaller and less stable (Goldsmith et al., 2016; Haapakoski et al., 2015; Osimo et al., 2020). In absolute terms, psychiatric IL-6 concentrations typically remain in the 2-4 pg/mL range (Roohi et al., 2021). By comparison, Damas et al. (1992) reported median IL-6 levels of 788 pg/mL in sepsis without shock and 10,049 pg/mL in septic shock. A recent cohort of 1669 patients with severe sepsis or septic shock reported median IL-6 of 772 pg/mL, 2137 pg/mL in non-survivors, and an approximate mortality-associated threshold of 15,000 pg/mL (Ruiz-Rodriguez et al., 2026). Thus, the order-of-magnitude gap between typical psychiatric IL-6 values and sepsis remains robust in contemporary data.

CRP shows similar moderate elevations across schizophrenia and depression meta-analyses, with values typically remaining in the low single-digit mg/L range (Fernandes et al., 2016; Haapakoski et al., 2015; Osimo et al., 2020). This is far below levels seen in acute bacterial infections (often >100 mg/L) and below many active systemic inflammatory diseases, while overlapping the chronic low-grade inflammatory range. For cardiovascular risk stratification, CDC/AHA high-sensitivity CRP bands classify <1 mg/L as low risk, 1-3 mg/L as average risk, and >3 mg/L as high risk (Pearson et al., 2003). Thus, the 2-4 mg/L range often observed in psychiatric cohorts can be clinically meaningful in a biologically defined context, even though it is not diagnostic for schizophrenia or depression. As with NfL and S100B, clinical value is unlikely to reside in stand-alone diagnostic cutoffs but in individual-level stratification. Baseline CRP has been reported to inform antidepressant selection (Jha et al., 2017). Whether IL-6 or TNF-alpha can serve analogous functions remains to be established.

Additional blood-based biomarker candidates are summarized in the Supplementary Table.

### 4.5 Signal specificity

Most inflammatory biomarkers studied in psychiatric disorders show cross-diagnostic rather than disorder-specific performance. For example, a patient with elevated IL-6 could meet criteria for schizophrenia, major depression, bipolar disorder, or an inflammation-related medical condition. Such cross-diagnostic signals may reflect shared pathobiology.

This pattern contrasts with markers in well-defined diseases, where markedly elevated NfL suggests neurodegeneration and extreme neutrophilia indicates infection (Al Shweiki et al., 2019; Eratne et al., 2024). Biomarker elevations in symptom-defined psychiatric disorders are typically milder and appear non-specific at the group level. However, this does not preclude their relevance within subgroups, where the same markers may reflect distinct and clinically meaningful biological processes. Cross-diagnostic signals do not negate clinical utility. For example, CRP should trigger further diagnostic work-up rather than confirming a single diagnosis, and is widely used for this reason.

The cross-diagnostic nature of individual inflammatory markers supports multi-marker profiling approaches (see Section 2.3), but such approaches are only as reliable as the individual assays they build on, underscoring the importance of the single-analyte quality standards outlined in Sections 1.1, 1.2.

### 4.6 Contextual judgment

It is important to match claims to effect size without overselling minor differences. Biomarker effects in psychiatry are typically small to medium at the group level. Changes such as a 20% increase in IL-6 or CRP may be biologically informative but rarely support diagnosis or individualized care without additional evidence.

Cross-disciplinary collaboration sharpens this judgment. For example, a 2 pg/mL increase in IL-6 may be marginal in a psychiatric disorder, whereas the same increase in serum NfL can represent a meaningful shift in a young patient with NfL reference values of 5–10 pg/mL. Evaluating such findings with neurologists, immunologists, or laboratory medicine specialists may aid accurate interpretation (Slade et al., 2023).

## 5 Publication bias and research resilience

Publication bias, the tendency for positive findings to be published more frequently than negative or inconclusive results, distorts the evidence base and poses particular risks in psychiatric biomarker research, where many studies are exploratory, effect sizes are small, and the boundary between signal and noise is narrow (Ioannidis, 2005, 2011; Mlinaric et al., 2017). In this setting, selective publication can make fragile biomarker narratives appear more convincing than they are.

### 5.1 Evidence of publication bias in psychiatry

Across the sciences, the proportion of published papers confirming a hypothesis increased from approximately 70% in 1990 to about 85% by 2007 (Mlinaric et al., 2017). In psychiatry, among 238 completed trials funded by the Stanley Medical Research Institute, 86 were positive, 152 negative, and 86% of the positive studies were published versus 53% of the negative ones (Bowcut et al., 2021). This imbalance inflates apparent efficacy and can sustain false-positive biomarker narratives.

A landmark FDA analysis reinforced this concern (Turner et al., 2008). Among 74 FDA-registered antidepressant studies, 31% were never published and all 37 positive studies reached journals versus only 11 of 36 negative ones. The published literature suggested 94% positive results, whereas FDA re-analysis showed only 51%, a 32% inflation of effect estimates.

Outcome switching and selective reporting can compound the problem of publication bias. One audit found that ∼70% of psychiatric studies had major discrepancies from their original protocols, including selective emphasis on secondary or post-hoc findings (Bowcut et al., 2021).

### 5.2 Funnel plots, small-study effects, and their consequences

Funnel plots provide visual diagnostics for publication bias (Egger et al., 1997). Asymmetric plots, particularly those missing small negative studies, suggest selective reporting (Sterne and Egger, 2001). An umbrella review of 162 peripheral biomarkers across five psychiatric conditions found that most meta-analyses had high heterogeneity and evidence of bias (Carvalho et al., 2020). When bias-adjustment methods such as trim-and-fill (Duval and Tweedie, 2000) or Egger's regression (Egger et al., 1997) were applied, many supposed effects became smaller or non-significant. The cumulative consequence is the amplification and canonization of false-positive effects that channel resources and careers toward misleading hypotheses. **Vignette 3** illustrates how hypothesis attachment and non-publication of null results can exacerbate this dynamic.

### 5.3 Research culture as a methodological safeguard

Publication bias is amplified when laboratories and institutions treat null results as personal or career failure. A healthy research culture treats rigorous positive and negative findings as equally informative, encourages preregistration and transparent reporting, and protects investigators from overinterpreting fragile effects simply because of the belief that positive narratives are easier to publish (Edmondson, 1999; Nosek et al., 2019).

### 5.4 Crucial negative results: the viral persistence study

The long-standing hypothesis that persistent brain viral infections contribute to serious mental illness illustrates the value of definitive negative evidence, which requires large cohorts and appropriate inferential frameworks (Dienes, 2014). A 2023 study performed whole-genome and RNA sequencing on post-mortem brain tissue from 1569 patients with schizophrenia, bipolar disorder, autism spectrum disorder, and controls (Min et al., 2023). Viral material was detected at low levels but showed no diagnostic differences, suggesting that chronic brain viral infection is unlikely to play a major role in these disorders. Publishing this result with editorial commentary (Steiner et al., 2023) exemplifies how negative evidence advances the field. Registered reports, preprints, and supportive journal policies offer practical tools to reduce publication bias more broadly (Nosek et al., 2019).

## 6 Quality checklist: safeguards for robust biomarker research

Robust psychiatric biomarker studies require explicit specification of intended use, rigorous analytical validation, and careful control of design, pre-analytical and statistical sources of bias (see Box 1).

Box 1 **Quality Safeguards for Robust Psychiatric Biomarker Research**

1. Define context of use (CoU)

Specify whether the biomarker is intended for diagnostic, prognostic, predictive, monitoring, pharmacodynamic, or safety-related use. Define the target population and clinical decision it informs and pre-register CoU and analysis plans to prevent post-hoc reinterpretation (Nosek et al., 2019).

2. Validate analytical performance

Establish assay validity across the relevant concentration range, including LOD and LOQ (e.g., CLSI EP17). Use blanks, low-concentration controls, and orthogonal validation (Pierson-Perry et al., 2017).

3. Ensure reproducibility and blinding

Blind laboratory staff to group status, include technical replicates, and confirm findings across independent batches or cohorts (Gavrielides et al., 2011; Hrobjartsson et al., 2013).

4. Control design and confounding

Use adequately powered, multi-site designs where possible, assuming small effects. Match or adjust for key confounders (age, sex, BMI, smoking, socioeconomic status), and standardize case-control collection (Simmons et al., 2011).

5. Standardize pre-analytical handling

Document and harmonize protocols for collection, processing, storage, and sample quality control across sites (Thachil et al., 2024; Toth et al., 2020).

6. Manage batch effects

Randomize samples across batches and reagent lots and use internal controls to detect drift and variation (Buhule et al., 2014; Stopsack et al., 2021).

7. Apply appropriate statistical inference

Predefine hypotheses, correct for multiple testing, report effect sizes with confidence intervals, and use appropriate models for distributional properties. Treat outliers cautiously and investigate biological plausibility before exclusion.

8. Avoid over-interpretation

Use precise language and avoid unsupported causal or diagnostic claims. Evaluate possible confounding and reverse causation.

9. Ensure transparency and reproducibility

Share data and code as possible and support independent replication and meta-analysis (Krauss et al., 2023).

10. Publish negative and null findings

Report well-conducted negative studies to reduce publication bias. Registered reports and preprints improve corrective feedback to the field.

Alt-text: Box 1

These safeguards are not exhaustive, but address common sources of irreproducibility and false discovery. Applied together, they can help bring psychiatric biomarker research closer to the standards established in other areas of medicine.

## 7 Conclusions

Distinguishing subtle biological signal from noise in psychiatric biomarker research is both a technical and translational challenge. Analytical limits, pre-analytical confounders, small effect sizes, and publication bias continue to shape the field. Much of the perceived diffuseness of psychiatric biomarker findings reflects the complexity of the underlying biology and the heterogeneity of the syndromic categories under investigation, rather than absence of biological underpinnings.

The key translational step is to stop treating weak average differences as failed diagnostics and instead test whether reproducible marker-defined subgroups differ in mechanism, prognosis, differential diagnosis, or treatment response. Multi-marker and multimodal approaches may improve subgroup identification, but only if they are analytically validated, replicated independently, and tested prospectively out-of-sample within a defined context of use. Pilot multimodal studies illustrate this frontier, including associations of serum nerve growth factor with regional gray-matter differences and serum BDNF with white-matter microstructure in schizophrenia (Hammans et al., 2020; Neugebauer et al., 2019).

Clinically useful biomarkers will emerge only through coordinated, multisite studies with reproducible methods, explicit contexts of use, and clinically interpretable thresholds. If accuracy is valued over allure and negative evidence is treated as knowledge, subtle and initially inconclusive findings can coalesce into dependable knowledge and, ultimately, better care for patients.

## Ethics approval and consent to participate

Not applicable.

## Consent for publication

Not applicable.

## Author perspective

The review originated from a lecture by JS and from the authors’ translational psychiatry, immunopsychiatry, proteomics, neurobiology, and clinical psychiatry experience. The bench vignettes are used as illustrative methodological examples rather than as primary empirical claims.

## Declaration of the use of generative AI and AI-assisted technologies in the preparation of this manuscript

The authors used the Deepl.com language editing tool. They reviewed and edited all material, and take full responsibility for the content of the article.

## Funding

JS and TNJ are PIs at the German Center of Mental Health (DZPG; Site Halle-Jena-Magdeburg: funding code 01EE2305A).

## CRediT authorship contribution statement

**Paul C. Guest:** Supervision, Writing – original draft, Writing – review & editing. **Janet L. Cunningham:** Conceptualization, Writing – review & editing. **Florian Raabe:** Writing – review & editing. **Kolja Schiltz:** Conceptualization, Writing – review & editing. **Leila Shokati Asl:** Data curation, Writing – review & editing. **Thomas Nickl-Jockschat:** Conceptualization, Funding acquisition, Writing – review & editing. **Johann Steiner:** Conceptualization, Funding acquisition, Project administration, Visualization, Writing – original draft, Writing – review & editing.

## Declaration of competing interest

The authors declare that they have no competing interests.

## Data Availability

No new data were generated or analyzed in support of this research. All data discussed are available in the cited publications.

## List of abbreviations

BD, bipolar disorder; CAP, community-acquired pneumonia; CSF, cerebrospinal fluid; CRP, C-reactive protein; FE-MDD, first-episode major depressive disorder; FE-SCZ, first-episode schizophrenia; HC, healthy controls; IL-6, interleukin-6; LOD, limit of detection; LOQ, limit of quantification; MDD, major depressive disorder; NfL, neurofilament light chain; NMDAR, N-methyl-D-aspartate receptor; SCZ, schizophrenia; SIMOA, single molecule array; TNF-α, tumor necrosis factor-alpha; bvFTD, behavioral-variant frontotemporal dementia.

## Vignettes

Lessons from the Bench (Vignette 1): Chasing Ghost Signals – the NMDAR antibody story In 2013, we screened sera from first-episode psychosis patients using a commercial cell-based immunofluorescence assay and observed a higher rate of low-titer NMDAR antibody positivity in patients than controls (Steiner et al., 2013). In two patients with high IgG titers and cerebrospinal fluid (CSF) confirmation, the work-up supported reclassification from primary psychosis to anti-NMDAR encephalitis, demonstrating genuine clinical value in rare cases. However, when we re-tested the same samples blindly using updated kit lots and stricter reading criteria, apparent group differences largely disappeared and control “positives” increased (Steiner et al., 2014b). Low-titer signals near the detection threshold in serum were unstable across runs and depended on assay format and subjective interpretation. Subsequent work emphasized that isolated low-titer serum antibodies can occur in healthy individuals, and that there is significant variation in antibody binding affinity and functional impact on the receptor. Current recommendations state that CSF testing and consideration of the clinical context are critical for interpretation (Steiner et al., 2020b). Personal account by corresponding author Johann Steiner.
**Lessons learned:**

• Treat near-threshold signals as provisional: define positivity thresholds and report test–retest reliability.
• Blind sample identity and replicate key assays; confirm findings with an orthogonal method when possible.
• Interpret serum autoantibodies in clinical context; use CSF and neurological “red flags” to avoid overdiagnosis.

Lessons from the Bench (Vignette 2): The S100B–BMI surprise S100B has often been discussed as an astrocyte-derived marker of glial pathology. In our work, we found that S100B is not astrocyte-specific in human brain, with expression across multiple cell types in and outside the brain (Steiner et al., 2007). Unexpectedly, we observed a strong association between serum S100B and BMI (Steiner et al., 2010a). Follow-up work showed peripheral sources and metabolic regulation of S100B, including links to insulin response (Steiner et al., 2014a). After adjusting for BMI and metabolic variables, apparent patient–control differences in S100B were markedly reduced, shifting our interpretation from “brain damage marker” toward a multi-system signal influenced by metabolic state. Personal account by corresponding author Johann Steiner.
**Lessons learned:**

• Measure and report variables such as BMI, smoking, and metabolic status, and test their impact on results. Present findings both with and without adjustment, as these variables may not only confound but also mediate the disease process. Correcting for them may remove the biological signal under investigation rather than clarify it.
• Be cautious with claims of cellular specificity; validate tissue sources and consider peripheral expression.
• Follow unexpected correlations: they may reveal hidden confounding or new biology.

Lessons from the Bench (Vignette 3): When hypothesis attachment outruns evidence Early in my career, I worked in a neurology laboratory testing the favored hypothesis of the workgroup leader that interferon-β (used to treat multiple sclerosis) induces neuronal apoptosis. Across multiple assays and conditions, results were consistently null. Instead of revising the hypothesis, we repeated variations without a clear stopping rule, and the negative data were never published. This experience illustrates how confirmation bias and flexible analytic or procedural choices can amplify noise and sustain false narratives (Born, 2024; Simmons et al., 2011). In contrast, later work in a research environment with higher psychological safety encouraged transparent discussion of negative results and facilitated learning and course-correction (Edmondson, 1999). Personal account by corresponding author Johann Steiner.
**Lessons learned:**

• Predefine hypotheses, primary outcomes, and stopping rules; treat well-conducted null results as informative.
• Design guardrails against confirmation bias (blinding, preregistration, independent replication).
• Choose (or build) lab cultures that reward rigor and openness, including publication of negative findings.
