# Supplementary material for: From case-control averages to validated subgroups: Interpreting inflammatory and neuroaxonal-injury biomarkers in schizophrenia and major depression
Source: Brain Behav Immun Health. 2026 Jun 8;55:101285. doi: 10.1016/j.bbih.2026.101285 (PMC13277546; doi:10.1016/j.bbih.2026.101285)
Supplement: Multimedia component 1 [file mmc1.docx]

**Supplementary Table.** Peripheral Blood-Based Biomarkers in Schizophrenia and Major Depression: Evidence Summary, Confounders, and Clinical Readiness

| **Biomarker(s)** | **Biomarker Category** | **Clinical Indication** | **Evidence — Schizophrenia** | **Evidence — Major Depression** | **Evidence Tier** | **Biomarker Type** | **Key Limitations & Confounders** | **Clinical Readiness** |
| --- | --- | --- | --- | --- | --- | --- | --- | --- |
| **INNATE IMMUNE MARKERS** | | | | | | | | |
| Absolute Neutrophil Count | Prognostic; Monitoring | Assess acute illness severity; predict exacerbation risk; monitor treatment response | Meta-analysis of 26,349 patients reports elevated neutrophils: first-episode g=0.85, antipsychotic-naïve g=1.17, overall g=0.69 (medium to large effects). Median counts remain within the reference range (~4.70×10⁹/L), but 23–30% of acute patients are above range vs. 6% of controls (Dudeck et al., 2025; Steiner et al., 2020). | Modest elevations in first-episode and recurrent MDD (g=0.37–0.51). Median counts remain within the reference range, with substantial overlap with controls; elevations less pronounced than in schizophrenia (Singh et al., 2022). | Tier 2 | State | Confounded by: acute infection, stress, smoking, obesity, corticosteroid use, antipsychotic type/dose. No validated clinical cutoff. Bacterial infection shows 2–4× higher counts (10–20×10⁹/L) vs. psychiatry. | NOT READY. Research use only. May assist acute inpatient severity assessment with additional clinical context, but lacks standardized protocols and clinical cutoffs. |
| Leukocyte Differential (Eosinophils, Monocytes, Lymphocytes) | Prognostic; Exploratory | Characterize immune cell profile subtypes; predict symptom clusters | First-episode psychosis: small elevations in eosinophils (g=0.31) and monocytes (g=0.26); medium increases in recurrent cases (g=0.39). Lymphocytes show heterogeneous responses (Dudeck et al., 2025; Steiner et al., 2020). | Limited data. Preliminary evidence of leukocyte shifts in inflammatory depression subtype. Heterogeneous findings across depression stages (Singh et al., 2022). | Tier 3 | State | Confounded by: circadian rhythm (eosinophils peak afternoons), medications (antipsychotics, antidepressants), acute stress, menstrual cycle. High inter-individual variability. | NOT READY. Exploratory biomarkers for understanding immune subtypes. Insufficient evidence for clinical stratification. |
| Neutrophil-to-Lymphocyte Ratio (NLR) | Prognostic; Monitoring | Predict acute exacerbation severity; correlate with symptom severity during acute phases; monitor response to antipsychotic treatment | Meta-analysis in non-affective psychosis shows elevated NLR vs. healthy controls (SMD ≈ 0.7; small–moderate group separation). Individual studies suggest associations with symptom severity (e.g., PANSS) and higher NLR in acute phase vs. remission (Mazza et al., 2020; Wang et al., 2024; Zheng et al., 2024). | Meta-analyses show that NLR is modestly elevated in major depressive disorder compared with healthy controls (SMD ≈ 0.33; small effect with substantial overlap), supporting low‑grade systemic inflammation but with limited diagnostic utility (Mazza et al., 2018; Su et al., 2022). | Tier 3 | State | Confounded by: acute infection, extreme stress, BMI, smoking status, time of day, menstrual cycle. Ratio methodology vulnerable to denominator effects (lymphopenia can artificially elevate NLR). No validated cutoffs. | NOT READY for routine use. Emerging utility in acute psychiatric inpatient settings but requires prospective validation studies and standardized protocols. |
| C-Reactive Protein (CRP) | Diagnostic; Prognostic; Predictive | Identify inflammatory phenotype; predict psychosis onset in ultra-high-risk (CHR) individuals; identify responders to anti-inflammatory augmentation | Meta-analyses show moderate CRP elevation in schizophrenia overall (g≈0.60), with larger effects in drug-naïve/free cohorts (g≈0.87) and first-episode samples (g≈0.63); absolute values generally remain in the low-grade inflammatory range. Mendelian randomization suggests low CRP may increase schizophrenia risk (reverse causation) (Fernandes et al., 2016; Hartwig et al., 2017). | Meta-analyses in major depression show moderate CRP elevation (d≈0.47 overall in cumulative analyses, with larger estimates in some broader or higher-quality syntheses), but values remain low-grade and highly overlapping with controls. CRP is among the more consistent markers in umbrella reviews, yet remains non-specific. High baseline CRP predicts poorer antidepressant response in some RCTs (Carvalho et al., 2020; Haapakoski et al., 2015). | Tier 1/2 | State/Trait (Mixed) | Confounded by: BMI, smoking, dietary factors, sedentary behavior, recent infection, metabolic syndrome, age, female sex, diurnal variation, season. No validated diagnostic cutoff for psychiatric use. | NOT READY for diagnosis. Clinical utility limited to research identification of inflammatory subtypes. May predict treatment response to anti-inflammatory agents in high-CRP subgroups, but no prospective RCT validation. |
| **INFLAMMATORY CYTOKINE PANEL** | | | | | | | | |
| Interleukin-6 (IL-6) | Diagnostic; Prognostic; Predictive | Identify inflammatory phenotype; predict treatment response; monitor disease activity | Most consistent cytokine signal. Acute meta-analytic effect sizes are largest in first-episode schizophrenia (≈1.16) and lower in acute relapse (≈0.73); chronic schizophrenia shows smaller persistent elevations (≈0.27). Absolute serum levels are typically 2–4 pg/mL in patients vs. ~1 pg/mL in controls (Goldsmith et al., 2016). | Elevated in depression vs. controls, but effect-size estimates vary across meta-analyses and inclusion criteria: 0.76 in acute MDD, g=0.61 overall (Osimo et al., 2020), and d=0.54 overall / d=0.60 in the high-quality subset; chronic MDD shows smaller persistent elevations (≈0.39) (Haapakoski et al., 2015). | Tier 2 | State | Confounded by: BMI (strong correlation), infection status, medications (SSRIs may reduce; NSAIDs are anti-inflammatory), acute stress, sleep deprivation, diurnal variation, and menstrual cycle.  CRITICAL: cytokine assays often operate near the limit of quantification (LOQ) in CSF. For comparison, sepsis IL-6 concentrations reach hundreds to tens of thousands of pg/mL, and much higher values have been reported in septic shock (Damas et al., 1992). | NOT READY for individual diagnosis. Exploratory associations only. May identify inflammatory depression subtype for anti-inflammatory adjunctive trials (NSAIDs, IL-6 antagonists), but cutoffs not validated. |
| Tumor Necrosis Factor-α (TNF-α) | Prognostic; Predictive | Predict symptom improvement with antipsychotics; predict negative symptom response; identify cognitive decline trajectory | Mean elevations are modest and less stable than IL-6 (acute first-episode ≈0.31; acute relapse ≈0.22; chronic schizophrenia ≈0.30). Some individual studies suggest associations with negative symptoms, cognition, or symptom change after antipsychotic treatment, but replication is inconsistent (Goldsmith et al., 2016; Lin et al., 2021). | TNF-α is modestly elevated in acute depression (overall d≈0.40), but the cumulative effect remains uncertain because of substantial heterogeneity and subgroup inconsistency; chronic MDD shows no significant mean difference. Baseline TNF-α has been linked to executive dysfunction in some studies, while evidence as a treatment-response predictor remains limited (Goldsmith et al., 2016; Haapakoski et al., 2015). | Tier 3 | State | Confounded by: BMI, smoking, physical activity level, metabolic syndrome, antipsychotic dose/type (some increase TNF-α acutely), age. Small sample sizes (often n<200); inconsistent replication. Sex differences inadequately characterized. Meta-analytic results are available (Goldsmith et al., 2016) but heterogeneity is substantial and subgroup effects are inconsistent. | NOT READY. Preliminary prognostic signal for negative symptom response, but requires prospective validation in larger RCTs before clinical implementation. |
| Interleukin-1β (IL-1β) | Diagnostic; Prognostic | Assess inflammatory burden; predict cognitive decline | Some meta-analytic evidence for elevated IL-1β in schizophrenia, but heterogeneity across studies is high. Associations with negative symptoms and cognitive deficits have been reported in individual studies but are not consistently replicated (Goldsmith et al., 2016). | Meta-analytic evidence for IL-1β elevation in major depression is inconsistent. The largest cumulative meta-analysis found no significant association (d = −0.05, p = 0.86). Individual studies report conflicting results (Goldsmith et al., 2016; Haapakoski et al., 2015). | Tier 2 | State | Confounded by: BMI, smoking, infection history, metabolic parameters. Most psychiatric studies do not measure IL-1β alongside IL-6/TNF-α; fragmented literature. | NOT READY. Exploratory inflammatory marker with inconsistent meta-analytic support; clinical utility not established. Requires integration into comprehensive inflammatory phenotyping. |
| **NEUROAXONAL INJURY MARKERS** | | | | | | | | |
| Neurofilament Light Chain (NfL) | Diagnostic; Prognostic; Monitoring | Differentiate psychiatric from neurodegenerative disease; monitor neurodegeneration trajectory; predict cognitive decline; assess neuroprotection during treatment | Psychiatric blood NfL findings in schizophrenia are generally small and inconsistent. Reported serum/plasma values are usually in the single-digit to low-teens pg/mL range and often overlap controls; differential-diagnosis cohorts mainly show much larger elevations only in neurodegenerative disease controls (Al Shweiki et al., 2019; Bavato et al., 2024; de Oliveira et al., 2025; Rodrigues-Amorim et al., 2020). | Meta-analysis reports WMD=8.78 pg/mL higher in depression vs. controls (95% CI: 5.28–12.28, p<0.01). Despite statistical separation, overlap with healthy controls remains substantial; differential-diagnosis value is stronger when psychiatric cohorts are contrasted with clearly neurodegenerative disease controls. NfL correlates with executive dysfunction severity in some studies (Bavato et al., 2024; Chen et al., 2022; Habibzadeh et al., 2024). | Tier 2 | Trait | Strongly age-dependent (exponential increase >60 years; requires age-adjusted interpretation). Confounded by: renal function, comorbid neurological disease, BMI, chronic kidney disease, brain injury history. Cost limits routine use. Requires ultra-sensitive platform (SIMOA, PEA); standard immunoassays have insufficient sensitivity. | NOT READY for psychiatry. Promising research biomarker for identifying occult neurodegeneration (especially elderly with cognitive decline). Strong utility in differentiation from primary neurodegenerative disease where NfL markedly elevated. Consider when cognitive decline unexpectedly prominent; refer to neurology if NfL markedly elevated. |
| **GLIAL / CNS DYSFUNCTION MARKERS** | | | | | | | | |
| S100B Protein | Diagnostic; Exploratory | Assess glial dysfunction and BBB permeability; exploratory marker of astrocytic/oligodendrocytic stress. | Earlier summaries suggested elevated peripheral S100B across schizophrenia and mood disorders, but heterogeneity is large and disease specificity is poor (Schroeter and Steiner, 2009). A more recent systematic review across five major psychiatric disorders concluded that most studies report elevation, but some report no difference or decreases (Kozlowski et al., 2023). When BMI is included as a covariate, the patient–control difference may attenuate or disappear; insulin resistance is also associated with S100B elevation (Steiner et al., 2010a; Steiner et al., 2010b). | Some studies show elevation, whereas others report no difference or decreases; overall the literature suggests only modest shifts with high heterogeneity, and proposed antidepressant-response associations remain unreplicated (Kozlowski et al., 2023; Schroeter and Steiner, 2009). | Tier 2/3 | State/Trait (Mixed) | CRITICAL CONFOUNDER: Strong correlation with BMI; elevated S100B is not specific to astrocytic damage. S100B is expressed in peripheral tissues (e.g., adipocytes, melanoma cells, ependymal cells, hepatocytes). Additional confounders include insulin resistance (HOMA‑IR), metabolic state, antipsychotic‑induced metabolic effects, and renal function (Steiner et al., 2007; Steiner et al., 2010a). | NOT READY. Too many metabolic confounders; limited glial specificity. Consider only in research context with comprehensive metabolic profiling. If elevated: assess for metabolic abnormalities first. |
| Glial Fibrillary Acidic Protein (GFAP) | Exploratory | Assess astrocytic-specific dysfunction (more specific than S100B) | Tier 4 evidence. Very limited psychiatric data; not routinely measured. Some neuroimaging/postmortem studies suggest astrocytic changes, but CSF and serum studies are sparse (de Oliveira et al., 2025; Mukherjee et al., 2024; Rodrigues-Amorim et al., 2020). | Tier 4 evidence. Few clinical CSF and serum studies in MDD suggest elevated GFAP and astroglial involvement, but data remain sparse and non‑replicated (Michel et al., 2021; Steinacker et al., 2021). | Tier 4 | Unknown | Currently measured primarily in neurodegenerative disease research. Emerging as more specific astrocyte marker than S100B but requires clinical psychiatric validation. | NOT READY. Exploratory marker with high specificity for astrocytes (vs. S100B's peripheral expression). Future potential in psychiatric research if assay sensitivity/availability improve. |
| **NEUROTROPHIC FACTORS** | | | | | | | | |
| Brain-Derived Neurotrophic Factor (BDNF) | Prognostic; Monitoring | Assess neuroplasticity; predict treatment response; monitor antidepressant efficacy | Network meta-analysis shows reduced serum BDNF in schizophrenia vs. controls. Lower levels associated with cognitive deficits and negative symptoms. Antipsychotic treatment may partially normalize BDNF (Fernandes et al., 2015; Zou et al., 2024). | Serum BDNF significantly lower in depression vs. controls across multiple meta-analyses. BDNF increases after antidepressant treatment, suggesting state marker utility. Proposed as treatment response biomarker (Molendijk et al., 2014; Zou et al., 2024). | Tier 2 | State | Confounded by: time of blood draw, physical exercise (acute increase), BMI, smoking, menstrual cycle, platelet count (BDNF stored in platelets; serum vs. plasma distinction critical). Not disease-specific; reduced across multiple psychiatric and neurological disorders. | NOT READY for individual diagnosis. Promising state marker for treatment monitoring in depression. Requires standardized collection protocols (serum vs. plasma, fasting, time of day) before clinical implementation. |
| **AUTOIMMUNE / ANTINEURONAL ANTIBODY MARKERS** | | | | | | | | |
| Anti-NMDAR Antibodies (serum IgG) | Diagnostic; Exploratory | Identify anti-NMDAR encephalitis misdiagnosed as schizophrenia; assess for immunotherapy-responsive psychosis | Initial studies reported serum NMDAR antibodies in ~10 % of acutely ill patients with an initial schizophrenia diagnosis vs. ~0.4 % of healthy controls (Steiner et al., 2013). Subsequent studies found low‑titer serum ‘positivity’ in similar proportions of patients and controls, indicating that low‑titer serum antibodies are unreliable as disease markers (Dahm et al., 2014; Steiner et al., 2014). In contrast, high‑titer IgG GluN1 antibodies in CSF, combined with typical neurological features and MRI/EEG abnormalities, are highly specific for anti‑NMDAR encephalitis; CSF titers are more informative than serum and correlate better with outcome (Gresa-Arribas et al., 2014; Titulaer et al., 2013). | No large systematic screening data; evidence limited to isolated case reports of anti‑NMDAR encephalitis presenting with prominent depressive symptoms and very small serological screening samples with unclear clinical significance (Moldavski et al., 2021; Rong et al., 2017). | Tier 3 (serum); Tier 2 (CSF) | State (acute illness) | Major confounders: Assay platform (cell-based, peptide immunoassay, live vs. fixed cells yield discrepant results). Interpretive subjectivity in immunofluorescence (inter-rater agreement poor for weak/borderline signals). Many low-titer serum positives are false positives or non-pathogenic autoimmunity. Requires CSF confirmation + neurological features for clinical significance (Brooks et al., 2020; Thouin et al., 2021). | SERUM: NOT READY. Low sensitivity/specificity; high false-positive rate. CSF + NEUROLOGY: READY for specialized use. High-titer IgG in CSF + clinical signs + imaging abnormalities diagnostic for anti-NMDAR encephalitis (warrants immunotherapy). Recommend orthogonal assays to reduce false positives. |
| **METABOLIC & ENDOCRINE BIOMARKERS (Safety monitoring; metabolic phenotyping)** | | | | | | | | |
| Fasting Glucose, Lipid Panel (Total Cholesterol, LDL, HDL, Triglycerides), BMI/Weight | Safety (antipsychotic-induced metabolic effects); Pharmacodynamic | Monitor antipsychotic-induced metabolic side effects; assess cardiometabolic risk; guide lifestyle/pharmacological interventions | Antipsychotic-induced metabolic changes are typically large (unlike many inflammatory biomarkers). Weight gain and adverse changes in glucose/lipid profiles vary by agent, with highest risk for clozapine/olanzapine and lower risk for aripiprazole (American Diabetes et al., 2004; Leucht et al., 2013). | Atypical antipsychotics used as augmentation for treatment-resistant depression (TRD) carry similar metabolic risks. Long-term antidepressant treatment can also be associated with weight gain, although effects are typically milder than atypical antipsychotics (Gafoor et al., 2018; Himmerich et al., 2015). | Tier 1 | State | Not confounders but direct medication effects. Success of monitoring depends on patient compliance with measurement, clinical follow-up, and lifestyle/pharmacological interventions. | ✓ READY & CLINICALLY IMPLEMENTED. Standard of care per multiple guidelines (APA, ADA, NCEP-ATP III criteria). Recommended monitoring: baseline, week 4, 12, then every 3–6 months. Fasting labs preferred (exclude 12 hrs food). |
| HOMA-IR; Fasting Insulin | Prognostic; Metabolic phenotyping | Assess insulin resistance as risk factor for metabolic syndrome and cognitive decline | Insulin resistance and impaired glucose tolerance are already present in antipsychotic‑naïve schizophrenia (small–medium effect sizes for fasting insulin and HOMA‑IR), and are further exacerbated by second‑generation antipsychotics such as olanzapine and clozapine (Newcomer et al., 2002; Pillinger et al., 2017). | Meta-analyses show increased insulin resistance (higher fasting insulin and HOMA‑IR) in depression; epidemiological data suggest a bidirectional association between insulin resistance/diabetes and depression risk. Despite this, HOMA‑IR is rarely included as an outcome in standard depression biomarker studies (Fernandes et al., 2022; Kan et al., 2013). | Tier 3 | State/Trait (Mixed) | Requires fasting state; not practical in acute psychiatric settings. Confounded by diet, exercise, baseline metabolic syndrome, BMI. Not specific to psychiatric illness; marker of cardiometabolic risk broadly. | EMERGING. May help identify depression subtype at high metabolic risk (vs. pure mood dysfunction). Not ready for routine psychiatric biomarker panels but relevant for personalized medicine approaches. |
| Cortisol (diurnal rhythm; awakening response) | Exploratory; Confounder | Assess hypothalamic-pituitary-adrenal (HPA) axis dysfunction; identify stress-responsive depression phenotype | Meta-analyses show altered cortisol responses to psychosocial stressors across the psychosis spectrum (Cullen et al., 2020) and elevated basal blood cortisol in first-episode psychosis (Hubbard and Miller, 2019). Evidence for disrupted diurnal rhythms and the cortisol awakening response varies across studies, and normalization with antipsychotic treatment is inconsistent (Bradley and Dinan, 2010). | HPA axis hyperactivity is well-established in depression, especially in melancholic and psychotic subtypes: meta-analysis shows higher rates of DST non-suppression in psychotic and melancholic depression, and multiple studies report elevated morning cortisol and blunted diurnal rhythms in a substantial subset of depressed patients (Iob et al., 2020; Nelson and Davis, 1997). | Tier 2/3 | State | Cortisol measurements are strongly confounded by sampling time, awakening time, sleep and circadian disruption, acute stress, medications (e.g. glucocorticoids, antipsychotics, estrogens), and endocrine comorbidities such as PCOS or Cushing’s; the diurnal variation is large, with several‑fold higher levels in the early morning than in the late evening, so strict timing standardization is essential (Liu, 2024; O'Byrne et al., 2021). | EXPLORATORY for endophenotyping. HPA axis dysfunction may identify depression subtype responsive to targeted interventions (e.g., glucocorticoid antagonists). Not ready for routine psychiatric diagnosis/treatment selection. |
| Thyroid-Stimulating Hormone (TSH); Free T4 | Safety; Diagnostic exclusion | Exclude thyroid dysfunction masquerading as mood/psychotic disorder; monitor antipsychotic effects on thyroid function | Tier 1 evidence (routine screening). Overt and subclinical hypothyroidism can present with mood/anxiety symptoms, and cognitive impairment; routine thyroid screening is therefore recommended in first-episode evaluations (Bauer et al., 2008). | Tier 1 evidence. Thyroid dysfunction (including subclinical hypothyroidism) is associated with depressive symptoms; meta-analyses report an association between hypothyroidism and depression (Bode et al., 2021; Loh et al., 2019). Lithium substantially increases the risk of hypothyroidism (elevated TSH) (Shine et al., 2015). | Tier 1 | Trait | Thyroid dysfunction is a medical comorbidity, not a disease-specific psychiatric biomarker. Confounded by age, sex, iodine intake, autoimmune thyroiditis (TPO antibodies), pregnancy, non-thyroidal illness, and medications (especially lithium) (Bauer et al., 2008; Shine et al., 2015). | ✓ ESTABLISHED STANDARD OF CARE. Essential for diagnostic exclusion; not a psychiatric biomarker. Baseline and periodic screening mandatory per clinical guidelines. Critical exclusion criterion in first-episode workup. |
| Prolactin | Safety; Monitoring | Monitor antipsychotic-induced hyperprolactinemia; assess for sexual dysfunction, osteoporosis risk, galactorrhea | Most antipsychotics (especially risperidone, paliperidone, haloperidol) elevate prolactin via D2 receptor blockade. Aripiprazole and quetiapine have lower prolactin-raising potential. Prevalence of hyperprolactinemia 40–90% depending on agent. Clinical consequences include sexual dysfunction, menstrual irregularity, galactorrhea, and long-term osteoporosis risk (Ghahramani and Bellon, 2024; Peuskens et al., 2014). | Same monitoring as in schizophrenia applies when antipsychotics are used as augmentation for treatment-resistant depression. | Tier 1 | State | Direct pharmacological effect, not a disease biomarker per se. Confounded by: pregnancy, pituitary adenoma, hypothyroidism, renal failure. Requires symptom screening at each visit (Ghahramani and Bellon, 2024). | ✓ EMERGING STANDARD OF CARE. NICE recommends prolactin monitoring at 6 months then annually for prolactin-raising antipsychotics. APA recommends symptom screening and level checking if clinically indicated. Not yet universally adopted as routine standard. |

**Evidence Tier Key:** Tier 1 – ≥3 meta-analyses, consistent, I²<50%, n>1,000, validated cutoffs | Tier 2 – Multiple large studies, some heterogeneity, n>500 | Tier 3 – Preliminary, n<500, high heterogeneity | Tier 4 – Single studies / case reports / theoretical basis only.

**References**

Al Shweiki, M.R., Steinacker, P., Oeckl, P., Hengerer, B., Danek, A., Fassbender, K., Diehl-Schmid, J., Jahn, H., Anderl-Straub, S., Ludolph, A.C., Schonfeldt-Lecuona, C., Otto, M., 2019. Neurofilament light chain as a blood biomarker to differentiate psychiatric disorders from behavioural variant frontotemporal dementia. J Psychiatr Res 113, 137-140.

American Diabetes, A., American Psychiatric, A., American Association of Clinical, E., North American Association for the Study of, O., 2004. Consensus development conference on antipsychotic drugs and obesity and diabetes. Diabetes Care 27, 596-601.

Bauer, M., Goetz, T., Glenn, T., Whybrow, P.C., 2008. The thyroid-brain interaction in thyroid disorders and mood disorders. J Neuroendocrinol 20, 1101-1114.

Bavato, F., Barro, C., Schnider, L.K., Simren, J., Zetterberg, H., Seifritz, E., Quednow, B.B., 2024. Introducing neurofilament light chain measure in psychiatry: current evidence, opportunities, and pitfalls. Mol Psychiatry 29, 2543-2559.

Bode, H., Ivens, B., Bschor, T., Schwarzer, G., Henssler, J., Baethge, C., 2021. Association of Hypothyroidism and Clinical Depression: A Systematic Review and Meta-analysis. JAMA Psychiatry 78, 1375-1383.

Bradley, A.J., Dinan, T.G., 2010. A systematic review of hypothalamic-pituitary-adrenal axis function in schizophrenia: implications for mortality. J Psychopharmacol 24, 91-118.

Brooks, J., Yarbrough, M.L., Bucelli, R.C., Day, G.S., 2020. Testing for N-methyl-d-aspartate Receptor Autoantibodies in Clinical Practice. Can J Neurol Sci 47, 69-76.

Carvalho, A.F., Solmi, M., Sanches, M., Machado, M.O., Stubbs, B., Ajnakina, O., Sherman, C., Sun, Y.R., Liu, C.S., Brunoni, A.R., Pigato, G., Fernandes, B.S., Bortolato, B., Husain, M.I., Dragioti, E., Firth, J., Cosco, T.D., Maes, M., Berk, M., Lanctot, K.L., Vieta, E., Pizzagalli, D.A., Smith, L., Fusar-Poli, P., Kurdyak, P.A., Fornaro, M., Rehm, J., Herrmann, N., 2020. Evidence-based umbrella review of 162 peripheral biomarkers for major mental disorders. Transl Psychiatry 10, 152.

Chen, M.H., Liu, Y.L., Kuo, H.W., Tsai, S.J., Hsu, J.W., Huang, K.L., Tu, P.C., Bai, Y.M., 2022. Neurofilament Light Chain Is a Novel Biomarker for Major Depression and Related Executive Dysfunction. Int J Neuropsychopharmacol 25, 99-105.

Cullen, A.E., Rai, S., Vaghani, M.S., Mondelli, V., McGuire, P., 2020. Cortisol Responses to Naturally Occurring Psychosocial Stressors Across the Psychosis Spectrum: A Systematic Review and Meta-Analysis. Front Psychiatry 11, 513.

Dahm, L., Ott, C., Steiner, J., Stepniak, B., Teegen, B., Saschenbrecker, S., Hammer, C., Borowski, K., Begemann, M., Lemke, S., Rentzsch, K., Probst, C., Martens, H., Wienands, J., Spalletta, G., Weissenborn, K., Stocker, W., Ehrenreich, H., 2014. Seroprevalence of autoantibodies against brain antigens in health and disease. Ann Neurol 76, 82-94.

Damas, P., Ledoux, D., Nys, M., Vrindts, Y., De Groote, D., Franchimont, P., Lamy, M., 1992. Cytokine serum level during severe sepsis in human IL-6 as a marker of severity. Ann Surg 215, 356-362.

de Oliveira, C.A., Pinto, J.P., de Pinho, C.S.N., Soares, M.V.R., Silva, M.F.S., Lucena, D.F., Braga-Neto, P., Campos, E.M., Sanders, L.L.O., Frota, A.F., Macedo, D.S., 2025. Blood-based biomarkers of neuroaxonal and astroglial injury identify ultra-treatment-resistant schizophrenia. Eur Neuropsychopharmacol 99, 16-18.

Dudeck, L., Nussbaumer, M., Nickl-Jockschat, T., Guest, P.C., Dobrowolny, H., Meyer-Lotz, G., Zhao, Z., Jacobs, R., Schiltz, K., Fernandes, B.S., Steiner, J., 2025. Differences in Blood Leukocyte Subpopulations in Schizophrenia: A Systematic Review and Meta-Analysis. JAMA Psychiatry.

Fernandes, B.S., Salagre, E., Enduru, N., Grande, I., Vieta, E., Zhao, Z., 2022. Insulin resistance in depression: A large meta-analysis of metabolic parameters and variation. Neurosci Biobehav Rev 139, 104758.

Fernandes, B.S., Steiner, J., Berk, M., Molendijk, M.L., Gonzalez-Pinto, A., Turck, C.W., Nardin, P., Goncalves, C.A., 2015. Peripheral brain-derived neurotrophic factor in schizophrenia and the role of antipsychotics: meta-analysis and implications. Mol Psychiatry 20, 1108-1119.

Fernandes, B.S., Steiner, J., Bernstein, H.G., Dodd, S., Pasco, J.A., Dean, O.M., Nardin, P., Goncalves, C.A., Berk, M., 2016. C-reactive protein is increased in schizophrenia but is not altered by antipsychotics: meta-analysis and implications. Mol Psychiatry 21, 554-564.

Gafoor, R., Booth, H.P., Gulliford, M.C., 2018. Antidepressant utilisation and incidence of weight gain during 10 years' follow-up: population based cohort study. BMJ 361, k1951.

Ghahramani, A., Bellon, A., 2024. Monitoring prolactin in patients taking antipsychotics. Front Psychiatry 15, 1333280.

Goldsmith, D.R., Rapaport, M.H., Miller, B.J., 2016. A meta-analysis of blood cytokine network alterations in psychiatric patients: comparisons between schizophrenia, bipolar disorder and depression. Mol Psychiatry 21, 1696-1709.

Gresa-Arribas, N., Titulaer, M.J., Torrents, A., Aguilar, E., McCracken, L., Leypoldt, F., Gleichman, A.J., Balice-Gordon, R., Rosenfeld, M.R., Lynch, D., Graus, F., Dalmau, J., 2014. Antibody titres at diagnosis and during follow-up of anti-NMDA receptor encephalitis: a retrospective study. Lancet Neurol 13, 167-177.

Haapakoski, R., Mathieu, J., Ebmeier, K.P., Alenius, H., Kivimaki, M., 2015. Cumulative meta-analysis of interleukins 6 and 1beta, tumour necrosis factor alpha and C-reactive protein in patients with major depressive disorder. Brain Behav Immun 49, 206-215.

Habibzadeh, A., Ostovan, V.R., Ghezel, M.A., Kavari, K., Kardeh, S., Tabrizi, R., 2024. Neurofilament light chain as a promising biomarker for depression diagnosis: a systematic review and meta-analysis. BMC Psychiatry 24, 617.

Hartwig, F.P., Borges, M.C., Horta, B.L., Bowden, J., Davey Smith, G., 2017. Inflammatory Biomarkers and Risk of Schizophrenia: A 2-Sample Mendelian Randomization Study. JAMA Psychiatry 74, 1226-1233.

Himmerich, H., Minkwitz, J., Kirkby, K.C., 2015. Weight Gain and Metabolic Changes During Treatment with Antipsychotics and Antidepressants. Endocr Metab Immune Disord Drug Targets 15, 252-260.

Hubbard, D.B., Miller, B.J., 2019. Meta-analysis of blood cortisol levels in individuals with first-episode psychosis. Psychoneuroendocrinology 104, 269-275.

Iob, E., Kirschbaum, C., Steptoe, A., 2020. Persistent depressive symptoms, HPA-axis hyperactivity, and inflammation: the role of cognitive-affective and somatic symptoms. Mol Psychiatry 25, 1130-1140.

Kan, C., Silva, N., Golden, S.H., Rajala, U., Timonen, M., Stahl, D., Ismail, K., 2013. A systematic review and meta-analysis of the association between depression and insulin resistance. Diabetes Care 36, 480-489.

Kozlowski, T., Bargiel, W., Grabarczyk, M., Skibinska, M., 2023. Peripheral S100B Protein Levels in Five Major Psychiatric Disorders: A Systematic Review. Brain Sci 13.

Leucht, S., Cipriani, A., Spineli, L., Mavridis, D., Orey, D., Richter, F., Samara, M., Barbui, C., Engel, R.R., Geddes, J.R., Kissling, W., Stapf, M.P., Lassig, B., Salanti, G., Davis, J.M., 2013. Comparative efficacy and tolerability of 15 antipsychotic drugs in schizophrenia: a multiple-treatments meta-analysis. Lancet 382, 951-962.

Lin, C., Chen, K., Yu, J., Feng, W., Fu, W., Yang, F., Zhang, X., Chen, D., 2021. Relationship between TNF-alpha levels and psychiatric symptoms in first-episode drug-naive patients with schizophrenia before and after risperidone treatment and in chronic patients. BMC Psychiatry 21, 561.

Liu, P.Y., 2024. Rhythms in cortisol mediate sleep and circadian impacts on health. Sleep 47.

Loh, H.H., Lim, L.L., Yee, A., Loh, H.S., 2019. Association between subclinical hypothyroidism and depression: an updated systematic review and meta-analysis. BMC Psychiatry 19, 12.

Mazza, M.G., Lucchi, S., Rossetti, A., Clerici, M., 2020. Neutrophil-lymphocyte ratio, monocyte-lymphocyte ratio and platelet-lymphocyte ratio in non-affective psychosis: A meta-analysis and systematic review. World J Biol Psychiatry 21, 326-338.

Mazza, M.G., Lucchi, S., Tringali, A.G.M., Rossetti, A., Botti, E.R., Clerici, M., 2018. Neutrophil/lymphocyte ratio and platelet/lymphocyte ratio in mood disorders: A meta-analysis. Prog Neuropsychopharmacol Biol Psychiatry 84, 229-236.

Michel, M., Fiebich, B.L., Kuzior, H., Meixensberger, S., Berger, B., Maier, S., Nickel, K., Runge, K., Denzel, D., Pankratz, B., Schiele, M.A., Domschke, K., van Elst, L.T., Endres, D., 2021. Increased GFAP concentrations in the cerebrospinal fluid of patients with unipolar depression. Transl Psychiatry 11, 308.

Moldavski, A., Wenz, H., Lange, B.E., Rohleder, C., Leweke, F.M., 2021. Case Report: Severe Adolescent Major Depressive Syndrome Turns Out to Be an Unusual Case of Anti-NMDA Receptor Encephalitis. Front Psychiatry 12, 679996.

Molendijk, M.L., Spinhoven, P., Polak, M., Bus, B.A., Penninx, B.W., Elzinga, B.M., 2014. Serum BDNF concentrations as peripheral manifestations of depression: evidence from a systematic review and meta-analyses on 179 associations (N=9484). Mol Psychiatry 19, 791-800.

Mukherjee, K., Guest, P.C., Schiltz, K., Meyer-Lotz, G., Dobrowolny, H., Borucki, K., Bernstein, H.G., Nickl-Jockschat, T., Relja, B., Steiner, J., 2024. Longitudinal analysis of astrocyte-derived protein levels in the blood of drug-naive and relapsed patients with schizophrenia. J Psychiatr Res 180, 301-306.

Nelson, J.C., Davis, J.M., 1997. DST studies in psychotic depression: a meta-analysis. Am J Psychiatry 154, 1497-1503.

Newcomer, J.W., Haupt, D.W., Fucetola, R., Melson, A.K., Schweiger, J.A., Cooper, B.P., Selke, G., 2002. Abnormalities in glucose regulation during antipsychotic treatment of schizophrenia. Arch Gen Psychiatry 59, 337-345.

O'Byrne, N.A., Yuen, F., Butt, W.Z., Liu, P.Y., 2021. Sleep and Circadian Regulation of Cortisol: A Short Review. Curr Opin Endocr Metab Res 18, 178-186.

Osimo, E.F., Pillinger, T., Rodriguez, I.M., Khandaker, G.M., Pariante, C.M., Howes, O.D., 2020. Inflammatory markers in depression: A meta-analysis of mean differences and variability in 5,166 patients and 5,083 controls. Brain Behav Immun 87, 901-909.

Peuskens, J., Pani, L., Detraux, J., De Hert, M., 2014. The effects of novel and newly approved antipsychotics on serum prolactin levels: a comprehensive review. CNS Drugs 28, 421-453.

Pillinger, T., Beck, K., Gobjila, C., Donocik, J.G., Jauhar, S., Howes, O.D., 2017. Impaired Glucose Homeostasis in First-Episode Schizophrenia: A Systematic Review and Meta-analysis. JAMA Psychiatry 74, 261-269.

Rodrigues-Amorim, D., Rivera-Baltanas, T., Del Carmen Vallejo-Curto, M., Rodriguez-Jamardo, C., de Las Heras, E., Barreiro-Villar, C., Blanco-Formoso, M., Fernandez-Palleiro, P., Alvarez-Ariza, M., Lopez, M., Garcia-Caballero, A., Olivares, J.M., Spuch, C., 2020. Plasma beta-III tubulin, neurofilament light chain and glial fibrillary acidic protein are associated with neurodegeneration and progression in schizophrenia. Sci Rep 10, 14271.

Rong, X., Xiong, Z., Cao, B., Chen, J., Li, M., Li, Z., 2017. Case report of anti-N-methyl-D-aspartate receptor encephalitis in a middle-aged woman with a long history of major depressive disorder. BMC Psychiatry 17, 320.

Schroeter, M.L., Steiner, J., 2009. Elevated serum levels of the glial marker protein S100B are not specific for schizophrenia or mood disorders. Mol Psychiatry 14, 235-237.

Shine, B., McKnight, R.F., Leaver, L., Geddes, J.R., 2015. Long-term effects of lithium on renal, thyroid, and parathyroid function: a retrospective analysis of laboratory data. Lancet 386, 461-468.

Singh, D., Guest, P.C., Dobrowolny, H., Vasilevska, V., Meyer-Lotz, G., Bernstein, H.G., Borucki, K., Neyazi, A., Bogerts, B., Jacobs, R., Steiner, J., 2022. Changes in leukocytes and CRP in different stages of major depression. J Neuroinflammation 19, 74.

Steinacker, P., Al Shweiki, M.R., Oeckl, P., Graf, H., Ludolph, A.C., Schonfeldt-Lecuona, C., Otto, M., 2021. Glial fibrillary acidic protein as blood biomarker for differential diagnosis and severity of major depressive disorder. J Psychiatr Res 144, 54-58.

Steiner, J., Bernstein, H.G., Bielau, H., Berndt, A., Brisch, R., Mawrin, C., Keilhoff, G., Bogerts, B., 2007. Evidence for a wide extra-astrocytic distribution of S100B in human brain. BMC Neurosci 8, 2.

Steiner, J., Frodl, T., Schiltz, K., Dobrowolny, H., Jacobs, R., Fernandes, B.S., Guest, P.C., Meyer-Lotz, G., Borucki, K., Bahn, S., Bogerts, B., Falkai, P., Bernstein, H.G., 2020. Innate Immune Cells and C-Reactive Protein in Acute First-Episode Psychosis and Schizophrenia: Relationship to Psychopathology and Treatment. Schizophr Bull 46, 363-373.

Steiner, J., Schiltz, K., Walter, M., Wunderlich, M.T., Keilhoff, G., Brisch, R., Bielau, H., Bernstein, H.G., Bogerts, B., Schroeter, M.L., Westphal, S., 2010a. S100B serum levels are closely correlated with body mass index: an important caveat in neuropsychiatric research. Psychoneuroendocrinology 35, 321-324.

Steiner, J., Teegen, B., Schiltz, K., Bernstein, H.G., Stoecker, W., Bogerts, B., 2014. Prevalence of N-methyl-D-aspartate receptor autoantibodies in the peripheral blood: healthy control samples revisited. JAMA Psychiatry 71, 838-839.

Steiner, J., Walter, M., Glanz, W., Sarnyai, Z., Bernstein, H.G., Vielhaber, S., Kastner, A., Skalej, M., Jordan, W., Schiltz, K., Klingbeil, C., Wandinger, K.P., Bogerts, B., Stoecker, W., 2013. Increased prevalence of diverse N-methyl-D-aspartate glutamate receptor antibodies in patients with an initial diagnosis of schizophrenia: specific relevance of IgG NR1a antibodies for distinction from N-methyl-D-aspartate glutamate receptor encephalitis. JAMA Psychiatry 70, 271-278.

Steiner, J., Walter, M., Guest, P., Myint, A.M., Schiltz, K., Panteli, B., Brauner, M., Bernstein, H.G., Gos, T., Herberth, M., Schroeter, M.L., Schwarz, M.J., Westphal, S., Bahn, S., Bogerts, B., 2010b. Elevated S100B levels in schizophrenia are associated with insulin resistance. Mol Psychiatry 15, 3-4.

Su, M., Ouyang, X., Song, Y., 2022. Neutrophil to lymphocyte ratio, platelet to lymphocyte ratio, and monocyte to lymphocyte ratio in depression: A meta-analysis. J Affect Disord 308, 375-383.

Thouin, A., Gastaldi, M., Woodhall, M., Jacobson, L., Vincent, A., 2021. Comparison of N-methyl-D-aspartate receptor antibody assays using live or fixed substrates. J Neurol 268, 1818-1826.

Titulaer, M.J., McCracken, L., Gabilondo, I., Armangue, T., Glaser, C., Iizuka, T., Honig, L.S., Benseler, S.M., Kawachi, I., Martinez-Hernandez, E., Aguilar, E., Gresa-Arribas, N., Ryan-Florance, N., Torrents, A., Saiz, A., Rosenfeld, M.R., Balice-Gordon, R., Graus, F., Dalmau, J., 2013. Treatment and prognostic factors for long-term outcome in patients with anti-NMDA receptor encephalitis: an observational cohort study. Lancet Neurol 12, 157-165.

Wang, K., Liu, S., Huang, D., Guan, X., Chen, N., Xiu, M., Liu, D., Huang, Y., 2024. Onset age moderates the associations between neutrophil-to-lymphocyte ratio and clinical symptoms in first-episode patients with schizophrenia. Schizophrenia (Heidelb) 10, 110.

Zheng, Y., Zhou, X., Chen, K., Fu, Z., Zhang, P., Zhu, Q., 2024. Neutrophil/lymphocyte ratio is increased in the acute phase of schizophrenia and regardless the use and types of antipsychotic drugs. BMC Psychiatry 24, 876.

Zou, Y., Zhang, Y., Tu, M., Ye, Y., Li, M., Ran, R., Zou, Z., 2024. Brain-derived neurotrophic factor levels across psychiatric disorders: A systemic review and network meta-analysis. Prog Neuropsychopharmacol Biol Psychiatry 131, 110954.
